# Juegos y prácticas como micropolíticas en las instituciones sociales del Estado

**DOI:** 10.18294/sc.2024.4815

**Published:** 2024-04-03

**Authors:** Hugo Spinelli

**Affiliations:** 1 Doctor en Salud Colectiva. Investigador, Instituto de Salud Colectiva, Universidad Nacional de Lanús, Buenos Aires, Argentina. hugospinelli09@gmail.com Universidad Nacional de Lanús Instituto de Salud Colectiva Universidad Nacional de Lanús Buenos Aires Argentina hugospinelli09@gmail.com

**Keywords:** Centros de Salud, Hospitales, Prisiones, Universidades

## Abstract

El objetivo de este ensayo es analizar el juego como un elemento central en las prácticas que construyen micropolíticas en las instituciones sociales del Estado. Los principales conceptos que se trabajan son: juego, prácticas y micropolíticas. El análisis se recorta a las instituciones de los campos sociales haciendo énfasis en el tamaño material. La hipótesis es que el tamaño de la organización es inversamente proporcional al desarrollo del juego en las instituciones. Esta discusión se da en un contexto marcado por un fuerte desapego a lo público y a lo estatal, lo cual no hace más que agravar las profundas desigualdades sociales, el nihilismo y la aporofobia, con una crisis de legitimidad de las instituciones públicas frente al avance de ideas no democráticas en gobiernos elegidos democráticamente en varios países de América Latina y de otros continentes.

## INTRODUCCIÓN

Me sería absolutamente imposible vivir si no pudiera jugar. Cuando digo jugar no me refiero a jugar con un trencito de juguete, sino a jugar en el sentido en que el hombre juega. Julio Cortázar^1^

En el cotidiano de las instituciones sociales del Estado, la razón moderna y el juego conforman dos orillas, entre las cuales transcurre el caudaloso río de la vida institucional, donde perecen ideas y voluntades que abrevan en la razón moderna, y se derrumban proyectos minuciosamente planificados. Gobernantes, gestores y trabajadores, desde la orilla de la razón, esperan construir el puente que consiga unir ambas orillas, mientras minimizan el valor del juego en las prácticas, que se dan en la otra orilla, donde las micropolíticas mantienen la real efectividad de cada institución social del Estado. 

Durante el siglo XX, la ausencia de una teoría organizativa que diera cuenta de la complejidad de las instituciones sociales llevó a naturalizar la aplicación de la teoría general de la administración (TGA), con sus lógicas y modelos organizativos. La TGA, al ser extrapolada a las instituciones sociales, impidió e impide entender lo que se hace, conformando situaciones “esquizoides” en las personas que integran esas instituciones y que conviven en lo formal con estructuras organizativas basadas en misiones y funciones, organigramas y normas rígidas del mundo industrial que nunca se cumplen; mientras en lo informal participan de juegos no explícitos, muy útiles en el hacer, pero que no se entienden desde la racionalidad que estructura a esos agentes. Matus sintetiza esa situación tan común en las organizaciones sociales con la frase “se planifica lo que no se hace, y se hace lo que no se planifica”^2^^,^^3^^,^^4^.

Los dos párrafos anteriores fundamentan de manera empírica y teórica el problema que trabajamos en este artículo, que se enfoca en las instituciones sociales del Estado, a las que Bourdieu engloba bajo el concepto de “la mano izquierda del Estado”^5^. 

Los principales conceptos que trabajamos son: juego, prácticas y micropolíticas. Entendemos el juego como una forma de libertad “fundante de la cultura”, basada en la idea del *Homo ludens*^6^, *y* como un “hilo conductor de la explicación ontológica” en términos de Gadamer^7^, en relación con los conceptos de deseo e inmanencia^8^^,^^9^^,^^10^^,^^11^. De Michel Foucault tomamos el concepto de prácticas, quien las define por la regularidad y la racionalidad que acompañan los diferentes modos del hacer^12^. El concepto de micropolíticas lo trabajamos siguiendo los desarrollos de Deleuze y Guattari para quienes las micropolíticas no saben de condiciones, son puro devenir en el plano de la inmanencia y de los vínculos, en tanto trabajo vivo^10^^,^^11^^,^^13^^,^^14^. Esos conceptos transformados en apuestas, tienen todo el potencial del acontecimiento^9^. 

Este ensayo continúa la reflexión sobre el tema del juego desarrollado en textos anteriores^14^^,^^15^, y su objetivo es analizar el juego como un elemento central en las prácticas que conforman las micropolíticas en las instituciones sociales del Estado^14^. La hipótesis que se plantea es que el tamaño de la organización es inversamente proporcional al desarrollo del juego. 

### El juego en la modernidad, otra omisión epistemológica

Tanto en la Antigüedad como en la Edad Media, los juegos constituyeron la vida social de las personas y sus procesos de socialización^16^. Es durante la modernidad que el juego quedó limitado a cuestiones recreativas de la infancia y por fuera de la idea del trabajo, perdiéndose así su dimensión lúdica, con graves consecuencias para las instituciones, sus trabajadores y los usuarios^17^. ¿Por qué si había registros desde tiempos remotos de la importancia del juego como fenómeno social y de socialización a partir del siglo XIX se lo limitó a la infancia? La respuesta hay que buscarla en la Revolución Industrial, que junto a la vocación imperial de Inglaterra y la moral victoriana constituyeron un nuevo orden, en el que el pensamiento económico instaló la idea de la utilidad^6^^,^^18^ y del trabajo a destajo^19^^,^^20^. En esa lógica, el juego, al ser considerado improductivo y una pérdida de tiempo, fue anulado, y su lugar lo ocuparon diferentes racionalidades que invalidaron lo lúdico, formulando modelos universales que, desdeñando las prácticas, redujeron la comunicación a lo escrito. 

El *Homo sapiens* pasó a representar lo racional y sus órdenes debían ser cumplidas por el *Homo faber,* quien las ejecutaba sobre un objeto sumiso, “la cosa”. En la modernidad, la Revolución Industrial reemplazó al trabajo artesanal, la máquina pasó a ser sinónimo de progreso, y se mecanizaron los procesos sociales, deshumanizando la naturaleza ontológica de lo social. En todo ello, el juego fue anulado.

La razón moderna se fundamentó en la relación entre lo pensante (res *cogitans)* y la cosa externa (res *extensa)*, buscando modelos mecánicos como solución a los problemas, modelos que se aplicaron a lo social desde el positivismo. La fábrica^19^ y el hombre máquina^20^ se constituyeron en ideologías dominantes, a las que debían subordinarse las prácticas, incluidas las sociales, entendidas como lineales, mecánicas y repetitivas. Así se anularon otras epistemologías y lo social se interpretó desde una lógica de relaciones sujeto-objeto, omitiendo la pregunta por el ser. Y en ese “olvido”, se anuló el lenguaje^21^*,* lo lúdico^6^, y lo relacional^22^, omisiones que terminaron por negar la otredad. 

Para Deleuze y Guattari, “el sujeto y el objeto dan una mala aproximación del pensamiento. Pensar no es un hilo tensado entre un sujeto y un objeto, ni una revolución alrededor de otro. Pensar se hace más bien en relación entre el territorio y la tierra”^23^. Las concepciones de la razón moderna dominaron y dominan los contenidos curriculares universitarios, los cuales, en general, son replicados por sus egresados, independientemente de su formación disciplinar, edad, género o identidad política. Es esa razón moderna la que deja en evidencia las insuficiencias epistemológicas de los sujetos para jugar el juego social. Esto se vuelve visible cuando se pasa del “debe ser” a la acción, y se enfrentan procesos relacionales basados en el lenguaje y el juego que, a lo sumo, pueden describir, pero no los pueden jugar. Ante esa realidad, tienden a recaer en lo instrumental, buscan herramientas que imaginan van a solucionar los problemas, y repiten la pregunta ¿cómo se hace?, ignorando que el “saber cómo” expresa un medio y no una cultura. Confundir esas dimensiones lleva a la cosificación del otro^24^. La búsqueda de herramientas para dar respuestas al ¿cómo hacer? remite a la idea de una totalidad explicativa propia de la modernidad, la cual anula al sujeto, al pensamiento, a la acción, a lo múltiple y a las incertidumbres que constituyen el juego social. Pese a los fracasos de la razón instrumental en solucionar los problemas sociales, los agentes no se plantean cambiar las preguntas e interrogarse, por ejemplo, acerca de los porqués y los para qué, de lo que hacen y de lo que piensan^25^^,^^26^^,^^27^. 

Las lógicas del “debe ser” que dominan la enseñanza en las universidades fracasan ante el juego social, lo cual abre las puertas para el ingreso de la ironía sartreana: “el infierno es el otro”, que pasa a ser la explicación dominante ante el fracaso^28^. La complejidad de lo relacional, del lenguaje, y de las conversaciones, tan importantes para las instituciones sociales, no encuentran sustento en la razón moderna, que solo se propone describir lo que observa y tomar esa descripción como “real”, como la única verdad posible, basándose en estudios, técnicas, y métodos descontextualizados que abrevan en el positivismo, y en diagnósticos inviables, ya que anulan la otredad, el juego y las conversaciones. 

La teoría general de la administración, desde sus inicios, entre fines del siglo XIX e inicios del siglo XX, no tardó en identificar la complejidad de los sujetos y sus relaciones y, en su devenir, incrementó las apuestas a las tecnologías vía la informática, la robotización y la inteligencia artificial para sustituir la complejidad que representan los colectivos de trabajadores y trabajadoras. 

A contramano de esa lógica, las instituciones sociales incrementaron significativamente el número de trabajadores, así como el tamaño de sus instituciones, en las que se aplicaron las mismas normativas y lógicas de la industria, ya que las instituciones sociales fueron consideradas por la TGA como una fábrica más^29^. 

Las premisas fundantes de la TGA fueron las de concebir a la persona trabajadora como *Homo faber,* interpretar a la organización como una pirámide, entender lo institucional como una combinación de lo racional y lo material, y concebir los lugares de gestión y de gobierno como espacios de alta concentración de poder y racionalidad. Sin embargo, el devenir de las instituciones de los campos sociales presentó diferencias radicales con la visión industrial de la TGA. Esas diferencias se basan en que los campos sociales están estructurados en lógicas relacionales, centradas en el lenguaje y el juego. No obstante lo anterior, las organizaciones sociales siguen siendo pensadas y concebidas como si estuvieran dominadas por lógicas sujeto/objeto desligadas del territorio. Esas ambivalencias no resultan gratuitas para trabajadoras y trabajadores, ni para las personas usuarias de esos servicios sociales. Las ideas dominantes no pueden aceptar que organizar es necesariamente reorganizar^30^ y refundar. 

## LA VUELTA DEL JUEGO

La anulación del juego fue objetada, durante el siglo XX, por distintos pensadores que marcaron su relevancia, como Jean Piaget, Melanie Klein, Donald Winnicott, Lev Vygotsky, Jacques Derrida y Pierre Bourdieu. 

El juego, en general, no es objeto de estudio, pero sí lo encontramos en muchos textos, en expresiones como: “*lo que está en juego*”, o “*entrar en el juego*”. En este apartado vamos a trabajar la idea del juego como forma de libertad fundante de la cultura^6^, como “el hilo conductor de la explicación ontológica”^7^ y en su relación con la inmanencia y el deseo^10^^,^^11^. 

### El juego como fundamento de la cultura

En 1938, Johan Huizinga^6^ recupera el juego como objeto de estudio para las ciencias, y en su libro *Homo ludens,* realiza “una genealogía de la cultura con relación al juego”, al cual entiende como:

…una acción u ocupación libre, que se desarrolla dentro de unos límites temporales y espaciales determinados, según reglas absolutamente obligatorias, aunque libremente aceptadas, acción que tiene su fin en sí misma y va acompañada de un sentimiento de tensión y alegría y de la conciencia de «ser de otro modo» que en la vida corriente.^6^

La idea del juego excede lo humano e incluye al mundo animal, produciendo una herida narcisista al *Homo sapiens*, a la vez que anula cualquier fundamento racional de lo lúdico^6^. Para Huizinga, es en el juego donde se aprende^6^, y es en ese hacer en el juego que radica el origen de cualquier cultura. ¿Por qué y para qué se juega? se interroga Huizinga, y responde que esa pasión y esa intensidad que se pone en el juego no tiene una explicación biológica y resulta inexplicable para la razón^6^. 

El juego se opone a lo serio, ya que lo serio es lo estructurado, y todo juego es, por esencia, una actividad libre, que produce la sensación de sentirse a gusto^6^. Huizinga encuentra que, en la entrega del sujeto al juego, este supera a las bromas, a la lógica y a lo biológico, y destaca que tampoco se lo puede entender como una forma mental^6^. Se juega porque se siente gusto al hacerlo, dado que se lo vive como una expresión de libertad, como algo que permite escaparse de la rutina. El juego no es expresión del desinterés, sino que es cultura, que suele ir más allá del interés, y está encerrada en sí misma con límites de tiempo y espacio^6^. 

Para Huizinga, el juego, a la vez que crea orden, es orden, y exige un orden para ser jugado:

…el juego oprime y libera, el juego arrebata, electriza, hechiza. Está lleno de las dos cualidades más nobles que el hombre puede encontrar en las cosas y expresarlas: ritmo y armonía. […] El juego pone a prueba las facultades del jugador: su fuerza corporal, su resistencia, su inventiva, su arrojo, su aguante y también sus fuerzas espirituales, porque, en medio de su ardor para ganar el juego, tiene que mantenerse dentro de las reglas, de los límites de lo permitido en él. […] Cada juego tiene sus reglas propias.^6^

La cultura se juega siempre bajo un acuerdo tácito de las reglas y, en la medida que el juego se mantenga puro, se mantendrá el carácter de lo lúdico. Pero cuanto más complejo es el juego, mayor es el nerviosismo y la incertidumbre para los jugadores sobre lo que está en juego^6^. El propio desarrollo de una cultura hace que el juego no se mantenga invariable y se produzca una cuasi imperceptible transición del juego a la esfera de lo sagrado, marcando la continuidad en una triple unión que se produce entre juego, fiesta y acción sacra^6^.

La potencia de lo lúdico erosiona las bases del raciocinio y la tradición. En términos de Huizinga, así como “casi todo lo abstracto se puede negar, por ejemplo: el derecho, la belleza, la verdad, la bondad, el espíritu, Dios y lo serio. Todo se puede negar, pero el juego no”^6^. 

### El juego como hilo conductor de la explicación ontológica

El título de este apartado se corresponde con uno de los capítulos de *Verdad y método*, de Hans-Georg Gadamer^7^. El juego ocupa un lugar relevante en su obra, y lo considera un proceso natural, no exclusivo de los humanos, ya que también juegan los animales y la naturaleza^7^. Para Gadamer, el juego nunca es un mero objeto, sino que existe para aquel que participa en él^7^. 

La palabra alemana *spiel* se traduce al español como juego, pero en su traducción pierde una serie de asociaciones semánticas que tiene en alemán, tal como la señalan los traductores del libro de Gadamer. El significado original en alemán de la palabra *spiel* es danza, lo cual se conserva en algunos términos compuestos como *spielmann* (juglar). También se lo asocia al mundo del teatro: una pieza teatral es un *spiel* (juego), los actores son *spieler* (jugadores) y la obra no se interpreta, sino que se juega (*wirdgespielt*)^7^. Gadamer señala que el juego va más allá de la conciencia de quién lo juega y, si bien remite a la experiencia del sujeto, se distingue del comportamiento subjetivo del jugador^7^. 

Para el *Homo sapiens*, el juego no constituye algo serio, lo vive como una distracción y considera que el juego no es más que eso, un juego. No obstante, el jugador que no toma en serio al juego y lo subvalora es considerado un aguafiestas^7^. ¿Cuándo el juego es juego?: “cuando el jugador se abandona del todo al juego”, responde Gadamer, para quien la definición de qué es el juego no debe buscarse en la reflexión del jugador, ni como expresión de la subjetividad, sino en “el modo de ser del juego como tal”, y para ello hay que eliminar la idea de que el juego existe en las racionalidades impuestas a las instituciones del campo social^7^.

Para Gadamer, la pregunta deber ser dirigida hacia “el modo de ser del juego como tal”, lo cual lo lleva a reconocer que “el sujeto del juego no son los jugadores”, sino que es el juego, el cual se manifiesta a través de los jugadores en “procesos lingüísticos que conforman juegos lingüísticos”^7^. La fascinación del juego reside justamente en ese salirse de la conciencia para entrar en un contexto de movimientos que desarrollan sus propias dinámicas. El juego remite a un movimiento sin objetivos, a un presente continuo, que se recrea en una constante repetición, sin importar quién lo realiza, al punto que se llega a prescindir de la persona cuando en un diálogo se expresa “algo está en juego”. Esa centralidad del juego, le permite al jugador entregarse a él, y desligarse de la iniciativa que “constituye el verdadero esfuerzo de la existencia”, lo cual vuelve entendible el impulso a la repetición en el jugador, y el temor a cambiar el juego^7^. 

El jugador solo requiere de un otro que juegue y que responda con sus contrainiciativas^7^. Ese otro, no necesariamente debe ser un jugador real, ejemplo actual de ello es la gamificación tecnológica de los juegos. Según Gadamer:

Todo jugar es un ser jugado. La atracción del juego, la fascinación que ejerce, consiste precisamente en que el juego se hace dueño de los jugadores. Incluso cuando se trata de juegos en los que uno debe cumplir tareas que él mismo se ha planteado, lo que constituye la atracción del juego, es el riesgo de si “se podrá”, si “saldrá” o “volverá a salir”. El que tienta así es en realidad tentado. Precisamente las experiencias en las que no hay más que un solo jugador hacen evidente hasta qué punto el verdadero sujeto del juego no es el jugador sino el juego mismo.^7^

El mundo del juego permanece cerrado al mundo de los objetos. Jugar siempre es representar, el jugador vive el juego como una realidad que lo supera. En el juego no hay referencias a los espectadores y, si el juego se convierte en competición, amenaza su carácter lúdico^7^.

El juego se transforma en la acción de jugar y, por lo tanto, lo que surge como imprevisto e improvisado puede volverse repetible, y hasta puede llegar a transformase en permanente. De allí el carácter constructivo del juego, que puede convertirse en obra. Estas ideas entran en conflicto con las ideas dominantes en las ciencias, que exigen entender el juego, y para ello le exigen al sujeto mantenerse fuera del juego, proceso por el cual se termina reemplazando lo lúdico por lo racional, creando expertos, comentaristas y críticos, que comparten el no jugar el juego. Pero el juego necesita jugadores y, en esa contradicción, la universidad y sus integrantes suelen quedar entrampados.

### Juego, inmanencia y deseo

Nietzsche jerarquiza el juego, en tanto actividad previa a la implementación de las formas, y con ello rompe con el esquema tradicional del conocimiento, ya que no hay un *a priori*, dado que el juego crea y se anticipa a las formas^16^. Para Nietzsche, el juego es un objeto teórico, un esquema interpretativo y un proyecto incondicionado, que remite al concepto de inmanencia, en tanto puesta en juicio de las tradiciones del pensamiento, de las costumbres, de las autoridades y de los razonamientos, a los cuales considera como adquiridos. En todo ello, Nietzsche no hace más que enfrentar al concepto de trascendencia como una realidad superior, dominante en el pensamiento kantiano^8^. 

Esta noción de inmanensia nietzschiana, también está presente en la cultura oriental, como fenómeno psíquico que no lo trasciende, ya que es inherente a él y va unido de un modo inseparable a su esencia, aunque racionalmente pueda distinguirse de ella. El plano de la inmanencia no dialoga con el “debe ser” de la racionalidad formulada desde el plano de la trascendencia. El plano de la inmanencia entra en juego en apuestas singulares o acuerdos para compartir horizontes de donde surgen las micropolíticas expresadas, muchas veces, bajo formas lúdicas^7^^,^^9^.

El juego, como parte del trabajo, construye el presente en el plano de la inmanencia, el cual es considerado por Deleuze, siguiendo a Spinoza y a Nietzsche, como una realidad superior al mundo platónico de las ideas. Deleuze ubica al juego en el plano de los acontecimientos, de las singularidades y de las intensidades^10^. 

### Todos los juegos, el juego

Las situaciones que viven en el día a día las trabajadoras y los trabajadores de los campos sociales en sus instituciones, suelen ser muy incómodas y desilusionantes, y con el tiempo, a la desilusión se suma el enojo. Esto los lleva a no implicarse, a desafiliarse de la institución, ya que piensan que eso que vivencian es un problema propio de la institución en la que trabajan, y que no tiene arreglo. Su propia racionalidad les impide entender la naturaleza del juego en el cual están inmersos, no pueden considerar que el juego es la cualidad ontológica del trabajo en esas instituciones y, por el contrario, exigen la racionalidad de una fábrica, realidad muy alejada de las instituciones sociales. 

Esas personas que trabajan en instituciones sociales piensan e imaginan la vida institucional desde lo racional, y no desde el juego. Gadamer considera que, en la relación entre el jugador y el juego, el jugador no sabe que lo sabe^7^. La pregunta que surge es: ¿dónde aprendió el juego?, la respuesta es muy sencilla, en las prácticas. 

El desconocimiento sobre la importancia del juego se puede transformar en conocimiento si se induce a los equipos a conversar y a pensar lo que hacen todos los días, pero dejando que hablen los pies, y evitando que en sus relatos intervengan las cabezas con sus conocimientos, o el sentido común señalando el “debe ser” y anulando las ricas y singulares experiencias del hacer, las que asociamos a la expresión “que hablen los pies”. Esa expresión, que usamos desde hace muchos años, la hemos encontrado en Jacques Lacan, quien en una conferencia dictada en el *Massachusetts Institute of Technology*, el 2 de diciembre de 1975, ante la pregunta de Noam Chomsky sobre su opinión acerca del pensamiento, Lacan respondió siguiendo la polémica existente entre ambos:

Pensamos que pensamos con nuestros cerebros, pero personalmente yo pienso con mis pies. Esa es la única manera por la que puedo entrar en contacto con algo sólido. En ocasiones pienso con mi cabeza cuando choco con algo. Pero he visto suficientes encefalogramas para saber que no hay indicios de pensamiento en el cerebro.^31^

En talleres que realizamos con trabajadoras y trabajadores de diferentes áreas sociales usamos una dinámica que consiste en que cada persona relate sus tareas diarias con minuciosidad, describiendo qué hace, cómo lo hace, y con quién lo acuerda. Y las personas puestas a narrar, a medida que avanzan, se van dando cuenta que sus relatos nada tienen que ver con los saberes de sus cabezas, ni con las normas de la organización, sino que son habladas por los pies, desde sus prácticas. Sus relatos las sorprenden y le provocan risas, se asombran el descubrir las contradicciones entre las ideas de sus cabezas y los juegos de sus pies que transgreden normativas y organigramas, pero hacen posible lo imposible. 

El relato anterior no será muy distinto al que podemos encontrar en personas a cargo de una dirección, o de la gestión de una institución social, si le solicitamos que relate el día a día de su trabajo, desde lo que hace (sus pies, las prácticas), y no desde lo que entiende que debiera hacer (su cabeza, los planes). Puestos a relatar, sería muy extraño que expresen que se basan en procesos planificados, programados evaluados y monitoreados, que acompañen el cumplimiento de órdenes con rendición de resultados ante sus superiores y de las personas que tiene a cargo. Por el contrario, es muy probable que sus relatos nos remitan a juegos, aunque no mencionen esa palabra, a resultados inciertos y, seguramente, resulte complejo entender cuál es el juego dominante. También es muy probable que sus relatos pasen por diferentes ejemplos que permitan asociarlos a una danza; a un parque de diversiones con autitos chocadores, tren fantasma o montañas rusas; a una olimpíada con carreras de relevo de 4x100 metros, donde la estafeta pocas veces logra pasarse; a competiciones de nado, pero desincronizado; a escenarios de lucha libre o tiro al blanco; a las artes escénicas con tragedias, comedias, tragicomedias, dramas o monólogos; o, simplificando, lo termine asociando al Altón Pirulero donde “cada cual atiende a su juego…”.

Lo que seguro reconocerán quienes relaten su día a día es que los escenarios en los cuales desarrollaron su juego no forman parte de su formación universitaria. También reconocerán que cuando llegaron al puesto, lo hicieron llenos de planes, metas y objetivos, los cuales tenían en común ignorar el juego social. Y que, con el tiempo, fueron descubriendo y entendiendo que todos juegan en un proceso continuo e incierto de gran aprendizaje, no exento de frustraciones, pero tampoco exento de alegrías. Tampoco supuso el carácter complejo de los problemas que tuvo que enfrentar y las secuencias caóticas que se presentaban en su agenda, desarmando lo planificado y generando urgencias, que no siempre eran tales. También descubrió lo complejo de las comunicaciones y que la Torre de Babel no es solo un relato bíblico. También aprendió que los milagros existen y, sin saber por qué o cómo, a veces los problemas se solucionaban sin su intervención^32^.

La racionalidad administrativa trata de imponerle a las organizaciones sociales formas de hacer siguiendo normas, las cuales anulan las conversaciones y el juego, desconociendo que esos equipos, cuando alcanzan el sentido y el significado de su tarea, transforman a la institución en una ludoteca. Los relatos de las personas que trabajan, dirigen o gestionan instituciones sociales son muy diferentes a las que lo hacen en instituciones de carácter industrial, en las que tiende a dominar lo racional. Esto no significa idealizarlas y pensarlas como ajenas al conflicto, sino reconocerlas como instituciones más previsibles que las sociales. 

## ADIÓS A LA PIRÁMIDE

El carácter ontológico del proceso de trabajo en las instituciones sociales es de tipo artesanal, ya que el mecanismo de coordinación se realiza por “ajuste mutuo” lo cual desencadena un efecto dominó sobre la lógica que estructura la TGA^13^^,^^14^. Lo cual impacta en la forma organizativa que pasa a conformarse como una burocracia profesional^33^, dado el alto poder que existe en la base de la organización -núcleos operativos-, donde radica el trabajo artesanal de las y los profesionales^33^. Los ejemplos más evidentes de burocracias profesionales, según Henry Mintzberg, lo constituyen las universidades, los hospitales, las escuelas, las empresas de producción artesanal, las instituciones orientadas a la investigación y las escuelas de arquitectura, entre otros^33^. La burocracia profesional impacta en las formas institucionales, dada la centralidad y relevancia que tienen en el trabajo, el lenguaje y las conversaciones, lo cual transforma en piezas de museo a los organigramas, y las misiones y funciones formuladas desde el funcionalismo. También cambian las formas de gestión y de gobierno, dado que las transformaciones anteriores hacen que gestionar y gobernar las instituciones sociales implique una singularidad y complejidad desconocida por la TGA para esos cargos directivos^2^^,^^29^^,^^32^. 

Esas dinámicas institucionales que se producen por fuera de lo racional son jugadas diariamente, y llevan a las instituciones sociales a fragmentarse cual archipiélagos, que tratan de ocultarse con denominaciones que remiten a la idea de una institucionalidad única, fuerte y consolidada bajo el nombre de escuela, colegio, universidad u hospital. Esos nombres no representan más que una frágil escenografía, que se descubre al ingresar a la institución y reconocer los diferentes juegos, jugadores, conflictos y alianzas en dinámicas imposibles de predecir, que llevan a que esos archipiélagos se multipliquen y se reconfiguren de maneras imprevisibles. Esto nos señala que, en las instituciones sociales, la pirámide está en la cabeza de los jugadores y no en sus juegos.

### La impronta fabril en la materialidad de la institución social

Las instituciones sociales adoptaron acríticamente la materialidad de la institución fabril en diferentes campos sociales, lo cual llevó a generar instituciones de gran tamaño. La pregunta que nos hacemos es ¿cuál es la ventaja de tener grandes instituciones en las áreas sociales? La hipótesis que sostenemos es que el tamaño de la organización es inversamente proporcional al desarrollo del juego en las prácticas, lo cual no debe simplificarse y pensar que reduciendo el tamaño de las organizaciones se resuelven todos sus problemas, ya que son instituciones hipercomplejas^34^^,^^35^, en las que el tamaño de la organización es un elemento central de esa complejidad, pero no el único.

La ambición de la “gran fábrica” se encuentra en diferentes campos sociales y sigue seduciendo a los actores políticos que quedan capturados por proyectos faraónicos e inauguraciones fastuosas, pero cuya efectividad social es muy cuestionable. 

Nos preguntamos: ¿es el destino inexorable de las instituciones sociales el convertirse en instituciones totales?, ¿nos animaremos a pensar e implementar otras institucionalidades?, ¿aceptaremos que las grandes instituciones anulan el juego?, ¿cuál es el problema de tener pequeñas instituciones a escala humana, que comprendan las diferencias y singularidades de sus poblaciones en vez de intentar homogeneizarlas?

Melossi y Pavarini^36^, en su libro *Cárcel y fábrica*, analizan cómo la cárcel adoptó un modelo institucional influenciado por la fábrica, que le permite operar como un instrumento de poder y control social, perpetuando la disciplina y su poder coercitivo^37^, consolidando el sistema carcelario como un lugar de intersección entre pobreza y raza^38^. 

El modelo de la gran institución social se encuentra también en los tristemente célebres institutos de menores, creados en Argentina en 1931, bajo la dependencia del Patronato Nacional de Menores y que, en 1972, Enrique Medina graficó en su novela *Las tumbas*, nombre puesto por los propios internados a los institutos de menores. Allí el autor relata con tintes autobiográficos su ingreso al instituto de menores cuando cursaba el segundo grado de la educación primaria, y su devenir por diferentes instituciones de minoridad durante diez años. Rodolfo Walsh, en el lanzamiento del libro expresó:

En este mundo no hay casi más salida que el tránsito de víctima a victimario a través de una larga cadena de simulación y sometimiento […] una sociedad putrefacta que encierra niños en campos de concentración […] un testimonio vigoroso y sorprendente sobre una categoría de presos sociales.^39^

Goffman, en su obra *Internados: ensayos sobre la situación social de los enfermos mentales y otros reclusos*, destaca cómo las instituciones psiquiátricas afectan la identidad y la interacción social de los individuos, transformándose en instituciones totales, a las que caracteriza por perder los límites entre los diferentes roles al interior de la institución^40^. Basaglia adjetiva a los hospitales psiquiátricos como una “institución negada”, considerándolos instituciones opresivas y deshumanizadas, a pesar de la existencia de estructuras físicas y organizativas que pretenden ser lugares de tratamiento y cuidado, cuando en realidad niegan las necesidades y los derechos fundamentales de las personas con padecimientos mentales. Estas instituciones, en su opinión, perpetúan la segregación, la estigmatización y el maltrato hacia los pacientes^41^.

Basaglia estaba convencido de que los manicomios eran instituciones que no se podían reformar y que era necesario acabar con ellos para devolverles la libertad a los pacientes y convertirlas en centros de apoyo, ya que hasta entonces habían sido lugares de encierro, de tratamientos inhumanos y medicación forzosa, proponiendo a la autodeterminación de las personas internadas para que se reintegren a una vida digna basada en su principal consigna: “la libertad es lo que sana”^41^. 

## LO GRANDE DE LAS PEQUEÑAS INSTITUCIONES

Frente a una realidad social que se ha ido complejizando, postulamos instituciones pequeñas en cuanto al tamaño, con una configuración rizomática, en función de resguardar las micropolíticas que humanizan la vida institucional^10^. Entendemos a las instituciones sociales del Estado como una red social, no tecnológica, sino humana, centrada en conversaciones sustentadas en el compromiso entre lo que se dice y lo que se hace^42^, con la construcción de acuerdos para compartir horizontes^7^, con una fuerte impronta de lo simbólico, y con evidencias objetivas del impacto de las acciones en el territorio.

Es necesario construir instituciones sociales del Estado a escala humana, alejadas de todo resabio industrial, y concebidas desde lógicas relacionales, con la importancia y la complejidad de lo relacional. Entre las cualidades que consideramos necesarias para configurar otra institucionalidad, destacamos la necesidad de integrar institucionalmente las políticas sociales en el territorio; jerarquizar los vínculos sociales y culturales; humanizar el cuidado; reconocer la diversidad de identidades, etnias y culturas; y promover gestiones colegiadas con la inclusión de los actores del territorio^43^^,^^44^^,^^45^^,^^46^^,^^47^.

Postulamos otras institucionalidades, no porque sea fácil lograr consensos sobre esta propuesta, sino porque consideramos que es muy necesario hacerlo, lo cual no es sinónimo de fácil. Las instituciones a escala humana favorecen los juegos, las prácticas y las micropolíticas. Pero si las instituciones crecen, indefectiblemente, comienzan a ser invadidas por procedimientos burocráticos, con la consiguiente despersonalización de los procesos que terminan por anular los juegos. Es entonces, cuando el juego se vuelve serio y, al desaparecer lo lúdico, se fortalecen dinámicas arbóreas con toques kafkianos que llevan a la desafiliación de lo público, no solo en los trabajadores, sino también en los colectivos del territorio que dejan de sentir a esas instituciones del Estado como propias y empiezan a escuchar cantos de sirenas que les prometen lo que no les darán, pero que tampoco les dan las instituciones públicas. Al retirarse el juego, se devalúa la jerarquía de lo relacional, aparecen las normas y se tecnifica la acción comunicativa^48^.

Hablamos de otras institucionalidades en lo social y no de nuevas institucionalidades, ya que en buena parte estaremos volviendo sobre experiencias que fueron ignoradas o desplazadas por el avance de los modelos industriales y tecnológicos que dominaron y dominan, el pensamiento sobre las instituciones y sus formas organizativas. Todo ese proceso implicará necesariamente la división de muchas de las instituciones existentes en otras más pequeñas, también la eliminación de algunas formas institucionales como los manicomios y geriátricos, sin que ningún trabajador pierda su fuente de trabajo, pero manteniendo firme el propósito de humanizar los vínculos y de crear sentidos de pertenencia entre los territorios y las instituciones. 

 ¿Y los niveles centrales?, deberán tener una fuerte reconfiguración que los lleve a abandonar sus *habitus* de planificadores y prescriptores del “debe ser”, para transformarse en instituciones más pequeñas, pero mucho más inteligentes y ágiles, concentradas en el monitoreo de las dinámicas y acciones territoriales, apoyadas en fuertes y dinámicos sistemas de información que, mediante análisis integrados, produzcan información sobre las acciones, los procesos y los resultados, los cuales deberán ser discutidos en el territorio con las poblaciones, desde miradas que integren lo social, y que superen el saber disciplinario con acciones que busquen efectividad a través de la comparación de resultados desagregados en diferente niveles locales y regionales, respetando siempre las singularidades territoriales^14^^,^^49^. Con todo ello se busca desencadenar procesos que permitan análisis, proyecciones e intervenciones sobre temas y problemas territoriales, realizando acciones compensatorias, y respetando las idiosincrasias en procesos de traducción de saberes^50^. Todo lo anterior debe ser parte de sistemas de petición y rendición de cuentas transparentes y de libre acceso^2^. 

Los cambios propuestos serán resistidos, ya que afectarán intereses políticos, económicos, gremiales y de las culturas institucionales. Esas transformaciones son fáciles de escribir en el papel, pero muy complejas de implementar, porque tampoco se resuelven con decretos o leyes. Por ello sostenemos que todo este proceso debe ser acompañado de una fuerte acción de publicización, que transforme el modelo de la macroinstitución social en un problema público que se debata socialmente^51^^,^^52^, con el propósito de ir construyendo consensos sobre otras institucionalidades. Aun así, el camino será muy arduo, ya que no se trata de una discusión científica, sino que se trata básicamente de una discusión política en la que hay fuertes intereses en juego.

### De escalas económicas a escalas humanas

En el mundo industrial, una forma de clasificar a las instituciones es sobre la base del tamaño (grandes, medianas y pequeñas)^29^, por el volumen de sus activos o por el número de empleados. Esas clasificaciones siguen escalas económicas que no debieran primar en las instituciones sociales ya que, como señala el refrán popular, “lo barato sale caro”. 

Hay una rica discusión sobre el tamaño de las instituciones. Desde la teoría crítica de la Escuela de Frankfurt, diferentes autores señalaron que el tamaño de las instituciones puede moldear la experiencia humana y la sociedad, y que las mega instituciones no solo afectan la efectividad y la estructura interna de esas instituciones, sino que afectan también la libertad, la identidad individual, la participación democrática y la dinámica social. Los principales integrantes de esa escuela analizaron la relación entre racionalidad, dominación y organización social, encontrando que la racionalización y la organización burocrática moderna conducen a formas de dominación y alienación. 

Adorno y Horkheimer argumentaron que las estructuras organizativas masivas generan un sistema de control que inhibe la libertad y la autenticidad humana, convirtiendo a los individuos en meros engranajes de la maquinaria social^53^. Herbert Marcuse señaló las consecuencias alienantes de la sociedad industrial a través de las formas organizativas modernas, cada vez más grandes y centralizadas, las cuales limitan la libertad individual y tienden a promover una mentalidad conformista y alienante, en la que los individuos se adaptan a roles predefinidos y pierden su capacidad de pensamiento crítico^54^. Para Habermas, el tamaño de una institución puede influir en la esfera pública y en la toma de decisiones, argumentando que las estructuras organizativas masivas pueden distorsionar la comunicación y dificultar la participación democrática, lo que afecta la formación de opiniones y la discusión pública^48^. 

Emst Schumacher, intelectual y economista alemán, integrante por dos décadas del Chief Economica Advisor del National Coal Board de Gran Bretaña, en su libro *Lo pequeño es hermoso: Economía como si la gente importara*^24^, publicado en 1973, critica la orientación de las sociedades centradas en lógicas económicas, camino que también siguió la ciencia y la tecnología, en desmedro de las personas. Uno de los capítulos tiene como título “Un problema de tamaño”, en el que analiza el crecimiento de las grandes empresas y cómo algunas de ellas han creado lo pequeño dentro de lo grande, tratando de lograr así un equilibrio entre libertad y orden, donde la libertad se expresa en pequeñas unidades y el orden en una unidad de organización global. Schumacher se pregunta:

¿Qué escala es la apropiada? Depende de lo que nosotros estemos tratando de hacer; el problema de la escala es hoy extremadamente crucial tanto en lo político como en lo social y en lo económico […] La idolatría del gigantismo, sobre la que ya he hablado, es posiblemente una de las causas y ciertamente uno de los efectos de la tecnología moderna […] ¿Cuál es el significado de democracia, libertad, dignidad humana, nivel de vida, realización personal, plena satisfacción? ¿Es ese un asunto de mercancías o de gente? Por supuesto es un asunto de gente. Pero la gente solo puede ser realmente gente en grupos suficientemente pequeños. Por lo tanto, debemos aprender a pensar en términos de una estructura articulada que pueda dar cabida a una variada multiplicidad de unidades de pequeña escala. Si el pensamiento económico no puede comprender esto es completamente inútil. Si no puede situarse por encima de sus vastas abstracciones, tales como el ingreso nacional, la tasa de crecimiento, la relación capital/producto, el análisis input-output, la movilidad de la mano de obra y la acumulación de capital; si no puede alzarse por encima de todo esto y tomar contacto con una realidad humana de pobreza, frustración, alienación, desesperación, desmoralización, delincuencia, escapismo, tensión, aglomeración, deformidad y muerte espiritual, dejemos de lado la economía y comencemos de nuevo. ¿Acaso no tenemos ya suficientes “señales de los tiempos” que indican que hace falta volver a empezar?^24^^)^

Las ideas contenidas en el párrafo anterior nos ayudan a sostener que las instituciones sociales debieran ser pequeñas, con una lógica de reproducción de carácter rizomático^10^, enfrentando con ello las ideas dominantes de los modelos fabriles adoptados erróneamente, pero no inocentemente por las áreas sociales, y que dieron pie a la existencia de grandes hospitales, escuelas, universidades, tribunales y cárceles, por citar los ejemplos más conocidos, que han dominado la institucionalidad de lo social que, en su hegemonía, desdeñó a la institución pequeña como expresión de baja calidad profesional y científica. 

Norbert Elías se hace la siguiente pregunta: ¿cuándo hay más conflicto en una organización?, y arriba a las siguientes conclusiones: cuando los trabajadores son muchos, cuando no se conocen y cuando no saben lo que hacen los otros^55^, evidenciando lo obvio, que no es nada más que reconocer que lo social es por esencia relacional. 

Aspiramos a instituciones pequeñas con un número limitado de trabajadores, más humanizadas que tecnificadas, y centradas en el cuidado del otro. Instituciones preocupadas en incorporar “jugadores” y no espectadores, relatores o comentaristas, y donde prime la dimensión lúdica de trabajar jugando y jugar trabajando. Construyendo juegos sin ganadores ni perdedores, donde el placer surja del jugar, donde prime la humanización sobre la cosificación, los derechos sociales sobre el mercado, el cuidar sobre el curar y el aprender sobre el enseñar. 

Tomamos la expresión “*Quillahue*” de la lengua mapudungún -que significa lugar que ayuda- para interrogarnos si esas otras institucionalidades de lo social podrán convertirse en lugares que ayuden.

### Las instituciones del campo de la educación

Enseñar y aprender son conceptos centrales para la educación, pero implican concepciones muy diferentes. El enseñar supone que hay alguien que sabe y otro que no y, por lo tanto, se le debe enseñar al que no sabe. Esa concepción de la educación es considerada por Paulo Freire como una educación bancaria, ya que concibe que se aprende desde la pasividad del estar en el aula, sentado en el banco. Situación muy diferente a la idea del aprender, que parte de la premisa de que todas las personas saben algo y que cada una encontrará el tiempo y la mejor forma para aprender, en un proceso mediado por la acción, que no desdeña el juego. Así, mientras el enseñar le asigna al docente el rol central, en el aprender, el docente acompaña la iniciativa del estudiante, que en su búsqueda aprende desde la pregunta, mientras trabaja y juega^56^^,^^57^.

John Dewey, en EEUU, entre las últimas décadas del siglo XIX y primeras del siglo XX, enfatizó el aprendizaje basado en la experiencia y la participación del estudiante en entornos pequeños, en los que la interacción entre estudiantes y docentes fuese lo más cercana y personalizada posible, destacando la importancia de la experiencia práctica y la interacción social^43^. Abraham Flexner desarrolló experiencias exitosas, también en EEUU, siguiendo los postulados propuestos por Dewey en el *college*^44^, y sostuvo que la calidad de la universidad no se medía ni por su tamaño, ni por el número de programas, ni de estudiantes^58^. Experiencias similares se dieron en los primeros años del siglo XX desde el anarquismo, liderado por Francisco Ferrer Guardia desde Barcelona^59^^,^^60^.

Diferentes teóricos de la educación resaltan la importancia de la interacción personalizada, la colaboración y el aprendizaje activo, los cuales son más fáciles de lograr en entornos educativos pequeños y centrados en los estudiantes^57^^,^^61^^,^^62^^,^^63^^,^^64^^,^^65^. Francesco Tonucci -pedagogo italiano- sostiene:

Paradójicamente, podríamos afirmar que tienen éxito en la escuela los que no lo necesitan. La escuela, que debería contribuir a introducir la igualdad entre los ciudadanos, por el contrario, alimenta las diferencias […] El profesor no es el saber sino el mediador del saber […] La escuela transmisiva supone que el niño no sabe y va a la escuela a aprender, mientras el maestro enseña a quien no sabe. Esa es una idea infantil, que piensa al niño como un vaso vacío, mientras el maestro vierte conocimientos que llenan al niño gradualmente. […] El niño sabe y es competente y va a la escuela para desarrollar su saber.^65^

Las instituciones educativas pequeñas pueden facilitar una atención más personalizada y con mayores posibilidades de interacción con los profesores, creando sentidos de pertenencia más fuertes y colaborativos y facilitando aprendizajes más prácticos y personalizados. Diferentes publicaciones actuales señalan los beneficios de optar por escuelas pequeñas^66^^,^^67^^,^^68^^,^^69^. 

### Otra hegemonía institucional en el campo de la salud

Schumacher plantea una serie de principios aplicables a las organizaciones:

El primer principio se denomina El Principio de Subsidiaridad o El Principio de la Función Subsidiaria. Una formulación famosa de este principio dice lo siguiente: “Es una injusticia y al mismo tiempo un mal grave y un atentado contra el orden el asignar a una asociación más grande y más alta lo que organizaciones más reducidas y subordinadas pueden hacer”.^24^^)^

Nos basaremos en ese primer principio, para sostener que la autoatención^70^^,^^71^, las otras racionalidades no médicas^72^^,^^73^ y los centros primarios de salud tienen capacidades de resolver al menos el 90% de los padecimientos de las personas. Esa evidencia científica fue generada por Kerr White, en 1961, en su trabajo de ecología de la atención médica^74^, con datos de población blanca de EEUU del período 1928-1931 y datos poblacionales de Inglaterra y Gales del período 1946-1950, el cual fue replicado desde entonces en numerosas publicaciones. En un análisis sobre estudios de ecología de la atención médica, que publicamos recientemente, basado en nueve investigaciones realizadas en EEUU, Japón, Canadá, Austria, Corea del Sur, Israel y Austria, entre los años 1996-2018 y a nivel país, encontramos resultados muy similares al trabajo de Kerr White^75^, lo cual señala una regularidad en un período de 90 años que debe ser analizada y discutida, ya que no se condice con el ideario medicalizado de las sociedades modernas. 

A los centros primarios de salud se les ha negado un carácter institucional que los jerarquice, utilizando para ello diferentes expresiones, en las que se conjugan miradas que lo infantilizan, con expresiones diminutivas, o nominaciones erráticas como: “la salita”, “la posta sanitaria”, “la sala de primeros auxilios”, “el centro periférico”, o “el dispensario”. Todas esas expresiones implican una subvaloración institucional e, indirectamente, una sobrevaloración de la figura del hospital, el cual tiene una nominación unívoca e indiscutible. Otra forma de deslegitimar a los centros primarios de salud fue obligar a los médicos generales o médicos de familia a hacer guardias solo en hospitales cuando, por su especialización, debieran hacer guardia en los centros primarios de salud. Todas esas cuestiones simbólicas y semióticas no deben soslayarse en el debate por la jerarquización y legitimidad de los centros primarios de salud. 

Durante el siglo XX hubo numerosas experiencias exitosas de centros primarios de salud, pero pocas de ellas perduraron en el tiempo, a diferencia de los hospitales que, en todos los lugares que se crearon, no solo se mantuvieron, sino que crecieron a niveles -la mayoría de las veces- injustificados, desde la perspectiva de la epidemiología de los servicios y sistemas de salud^76^. 

Evidencias sobre la importancia y utilidad de los centros primarios de salud contiguos a las poblaciones se encuentran en EEUU, entre fines del siglo XIX e inicios del siglo XX^77^. Winslow, en 1919, afirmó que los centros primarios de salud constituían el acontecimiento más notable en la evolución de la salud pública de EEUU^77^. Michael Davis, en 1927, pronunció afirmaciones en el mismo sentido^14^^,^^78^. 

El *Dawson Report*^79^, publicado en Inglaterra en el año 1920, es considerado un documento central para lo que luego sería el *National Health Service*(NHS). Allí se hace referencia a tres niveles institucionales: centros primarios de salud, centros secundarios de salud (hospitales), y hospitales escuelas, que tienen funciones docentes. Hace más de 100 años, el informe Dawson estableció lo que debía componer un centro de salud primaria, su lectura no dejará de sorprender al lector:

En los centros primarios de salud se concentrarían las actividades y los servicios de salud de los distritos atendidos […] Habría salas de diversos tamaños y para distintos fines, incluso para obstetricia. Se tendrá en cuenta el empleo creciente de la fisioterapia natural de las enfermedades. […] Podría preverse además acomodación para: Quirófano, con el equipo necesario. Salas de radiología. Laboratorio para investigaciones sencillas. Dispensario. Baños, incluso para hidroterapia sencilla. Equipo necesario para masajes, electricidad, educación física […] Servicios para la comunidad […] Se contaría con locales para servicios de la colectividad tales como atención prenatal, bienestar de la infancia, inspección médica y tratamiento de escolares, educación física, examen de casos sospechosos de tuberculosis y enfermedades profesionales, etc. [...] Cuando en determinados distritos no se disponga por el momento de los servicios de parteras, el centro debería proporcionar tales servicios y además contar con locales para residencia, no solo de las enfermeras y parteras que allí trabajan, sino también para aquellas personas que prestan servicios análogos en la vecindad. […] Un aspecto importante del centro sería una clínica dental, con personal de cirujanos dentistas visitantes, empleados a tiempo parcial o, en caso necesario, a tiempo completo, y enfermeras adscritas al servicio […] El centro primario sería la sede de la organización de la salud y de la vida intelectual de los médicos de esa unidad. Los profesionales, en vez de estar aislados, como sucede ahora, podrían reunirse y estarían en relación con consultores y especialistas; se fomentaría así una corriente intelectual y una camaradería de gran provecho para el servicio. Con el tiempo se organizarían sin duda debates y cursos para graduados y sería fácil y provechoso otorgar licencias con fines de estudio en los hospitales docentes. Proporcionaría todas las formas ordinarias de tratamiento a pacientes de todas las edades [...] la labor del médico general seria principalmente domiciliaria y solo en parte institucional, en su mayoría individual, pero en parte pública.^79^

En 1946, John Grant, funcionario de la Fundación Rockefeller, planteó que el centro de salud del futuro se estaba por crear, en alusión a los centros primarios de salud^14^^,^^77^. En 1952, Henry Sigerist, en una conferencia en la Escuela de Higiene y Medicina Tropical de la Universidad de Londres sostuvo la importancia y necesidad de los centros primarios de salud^80^^,^^81^. 

En América Latina hay una larga lista de experiencias de diferentes magnitudes y en diferentes momentos y países pero que, en general, son de vida efímera. Esas experiencias son el producto del pensamiento de personas, del ámbito profesional o político, con valores humanistas, o de situaciones políticas en procesos revolucionarios.

En las últimas décadas, sobre todo en los países escandinavos, se ha logrado avanzar en la transición del hospitalocentrismo al centro primario de salud^82^^,^^83^. 

El Informe Dawson, a 104 años de su publicación, es una clara evidencia del grave error institucional que se llevó adelante al jerarquizar los centros secundarios de salud (hospitales), sobre los centros primarios de salud primaria. 

En el año 2017, Henry Mintzberg, en su libro sobre los mitos de la gestión en salud, señala que:

En las instituciones sanitarias, especialmente en los hospitales, la escala recibe considerable atención por parte de los ingenieros administrativos, que se ocupan de los problemas tratando de hacerlos más grandes […] A medida que las instituciones crecen, superponen un nivel de administración sobre otro en sus jerarquías formales. Ya hemos discutido lo que se puede llamar la política de escala: la inclinación a fusionar hospitales pequeños en otros más grandes por el poder de las administraciones. Esto también puede equivaler a la conveniencia de la escala: a las administraciones centrales les lleva tiempo y esfuerzo tratar con numerosas instituciones pequeñas: horas y horas de su tiempo. Cuánto más conveniente es agruparlos todos y ocuparse de uno solo, incluso si eso puede hacer que muchos proveedores de servicios se sientan miserables durante años. Cada uno de ellos, al estar cada vez más alejado de las operaciones, recurre cada vez más a la reorganización, la reingeniería, la medición y otras soluciones fáciles. […] Además, la gran escala puede imponer cierta conformidad a las actividades. Esto puede hacer que sean más fáciles de administrar, pero también puede sofocar la necesidad de adaptación local. […] Las instituciones pequeñas a menudo pueden abordar ciertos problemas de maneras menos formales y más efectivas. Para casi todo lo relacionado con la atención sanitaria (y fuera de ella), el imperativo de que la escala, la medición, el liderazgo, etc., sea una especie de “única mejor manera” es simplista y disfuncional. Viajar por la línea de montaje médico no es un chasis ni un motor, somos nosotros mismos” […]. Especialmente cuando estamos enfermos, pero incluso cuando estamos bien, a menudo encontramos que las grandes instituciones son impersonales y alienantes. Eso puede influir en la eficacia de los tratamientos que recibimos. No es de extrañar que se haya producido un alejamiento de los grandes hospitales, al igual que de las grandes escuelas. Para lograr una calidad real en la atención sanitaria, necesitamos servicios personalizados a escala humana, no intervenciones impersonales a escala económica. No estoy diciendo aquí que lo pequeño siempre es hermoso, solo afirmo que lo más grande no es necesariamente mejor […] El problema de la escala parece aplicarse también a la investigación farmacéutica. Algunas de las empresas más grandes se enfrentan desde hace años a dificultades en sus investigaciones. Un director ejecutivo de Merck, la empresa hasta ahora más reconocida por sus investigaciones, comentó que “la escala no ha sido un indicador de la capacidad de descubrir fármacos innovadores. De hecho, ha sido al revés: uno se estanca” […]. Gran parte de la investigación farmacéutica interesante proviene ahora de empresas más pequeñas.^84^

### La institución carcelaria

La cárcel, en su institucionalidad clásica, tiene bajas probabilidades de producir reinserción social^85^. La discusión sobre el tamaño de las prisiones ha sido objeto de atención por parte de académicos, activistas y profesionales del sistema judicial, quienes se interrogaban si las prisiones más pequeñas pueden ser más efectivas para la rehabilitación, al permitir, teóricamente una mayor atención individualizada, programas de reeducación más adaptados y relaciones más cercanas entre el personal y los reclusos. Las cárceles más pequeñas podrían ser más costosas, desde la lógica de la economía de escala; sin embargo, los costos adicionales pueden valer la pena si conducen a mejores resultados de rehabilitación y menores tasas de reincidencia. Múltiples voces^86^^,^^87^^,^^88^^,^^89^ argumentan a favor de un enfoque más centrado en el individuo y menos dependiente de grandes instalaciones, enfatizando la calidad de la rehabilitación sobre la cantidad de reclusos.

## ¿FINAL DEL JUEGO U OTRAS INSTITUCIONALIDADES?

Desde hace décadas asistimos a momentos históricos dominados por una primacía de lo individual y lo privado, en desmedro de lo colectivo y de lo público. Esa situación señalada desde la segunda mitad del siglo XX por diferentes autores^90^^,^^91^^,^^92^^,^^93^^,^^94^^,^^95^, fue una tarea que profundizó el neoliberalismo a nivel mundial en las últimas décadas, y que se expresó y expresa en gobiernos, en diferentes países, de características antidemocráticas, que tienen como objetivo acabar con los derechos sociales, y las instituciones públicas en nombre de la libertad del mercado. 

En las últimas décadas surgieron nuevas subjetividades que descreen de las instituciones sociales del Estado y de la idea de Nación. Nuevas subjetividades que también se reprodujeron en sectores sociales históricamente abandonados por las políticas de Estado y que padecen situaciones de exclusión social o pobreza extrema^96^. 

La dimisión o la retirada del Estado, la cosificación de los sujetos, la construcción de subjetividades autocentradas, la exclusión social y la crisis de legitimidad de lo público, configuran una realidad muy compleja, que se retroalimenta con el progresivo crecimiento de la deuda social. 

Los horizontes neoliberales en las subjetividades que avizoraba Jorge Alemán^94^, en menos de diez años se volvieron realidades políticas muy graves en Argentina. Parecen muy lejanos los debates sobre la democracia, el Estado y la ciudadanía, que se dieron en América Latina a partir de la década de 1980^97^^,^^98^^,^^99^^,^^100^. Los tiempos actuales nos señalan que esos temas aun constituyen deudas significativas en las sociedades latinoamericanas, deudas que no se pudo o no se quisieron saldar.

La ruptura de los lazos sociales, el dominio de la aporofobia, y el abandono de todo proyecto de reducción de inequidades interpelan a las instituciones sociales del Estado, a sus políticas y a las acciones en los territorios. Bourdieu, en la década de 1990, en Francia, dejó testimonios sobre la dimisión del Estado^96^, expresada en la ausencia del derecho al Estado y a la ciudadanía de sectores mayoritarios de la población, y postuló el concepto de “la mano izquierda del Estado” para señalar cómo la pauperización de amplios sectores sociales en Francia complejizaba el trabajo de las instituciones sociales del Estado y el impacto de esas nuevas realidades en las subjetividades.

Frente a esa realidad, resulta imperativo colocar en agenda la necesidad de producir cambios culturales y estructurales en las instituciones sociales del Estado, a los fines de poder recuperar su función social y brindar servicios públicos de calidad, desde los territorios. Es allí, en los territorios, que esas instituciones son una de las “caras” del gobierno, es decir, se transforman en importantes intermediarios entre los gobiernos y la población. Revertir esas caracterizaciones excede, en parte, las posibilidades de quien presida un gobierno nacional, provincial o municipal, ya que las áreas sociales, por su propia características de burocracias profesionales^33^ pueden producir milagros, o quedar autocentradas con desafiliación a lo público y boicotear las políticas originadas en niveles centrales. 

El desafío que enfrentan quienes gobiernan, quienes trabajan en las instituciones públicas y las numerosidades sociales en los territorios, es cuidar esa institucionalidad que debe priorizar derechos, sobre todo, en quienes más los necesitan, y para ello tienen que brindar el mejor servicio posible. Tanto funcionarios, como trabajadores debieran recuperar con orgullo el concepto de servidores públicos, para generar una cultura del trabajo que dé cuenta de la otredad de los usuarios. De nada sirve sentirse militante de lo social si se mantienen prácticas centradas en el “debe ser”, se niega lo lúdico y la otredad. 

No debemos aceptar el fin del juego, hay que habilitar institucionalidades pequeñas, donde el juego construya micropolíticas en las prácticas que acompañen y cuiden a quienes más lo necesitan.

¡A jugar!
