# Supplementary material for: Juegos y prácticas como micropolíticas en las instituciones sociales del Estado
Source: Salud Colect. 2024 Apr 3;20:e4815. [Article in Spanish] doi: 10.18294/sc.2024.4815 (PMC11822900; doi:10.18294/sc.2024.4815)
Supplement: Translation

# Translation: Plays and practices as micropolitics in State social institutions

**Authors:** Hugo Spinelli

**Affiliations:** 1 Doctor in Collective Health. Investigator, Instituto de Salud Colectiva, Universidad Nacional de Lanús, Buenos Aires, Argentina. nnvalencia@correo.unicordoba.edu.co

**Keywords:** Health Centers, Hospitals, Prisons, Universities

## Abstract

The aim of this essay is to analyze plays as a central element in the practices that construct micropolitics within the social institutions of the State. The main concepts addressed are: plays, practices, and micropolitics. The analysis focuses on institutions within social fields, emphasizing material size. The hypothesis posits that the size of the organization is inversely proportional to the development of plays within the institutions. This discussion takes place in a context marked by a strong detachment from the public and the state, exacerbating profound social inequalities, nihilism, and aporophobia, alongside a crisis of legitimacy of public institutions in the face of the advance of non-democratic ideas in democratically elected governments in several countries in Latin America and other continents.

## INTRODUCTION

“I would find it absolutely impossible to live if I couldn’t play. When I say play, I don’t mean playing with a toy train, but playing in the sense in which a man plays.” - Julio Cortázar.^1^

In the everyday life of State social institutions, modern reason and play constitute two shores, between which the mighty river of institutional life flows, where ideas and wills that are nourished from modern reason perish, and meticulously planned projects, collapse. Governors, managers, and workers, from the shore of reason, hope to build the bridge that can connect both shores, while minimizing the value of play in practices, which occur on the other shore, where micropolitics maintain the real effectiveness of each social institution of the State.

During the 20th century, the absence of an organizational theory that accounted for the complexity of social institutions led to the naturalization of the application of general management theory (GMT), with its organizational logics and models. GMT, when extrapolated to social institutions, prevented and prevents understanding what is being done, creating “schizoid” situations in the people who integrate these institutions and who coexist formally with organizational structures based on missions and functions, organizational charts, and rigid rules from the industrial world that are never fulfilled; while informally they participate in non-explicit plays, very useful in doing, but which are not understood from the rationality that structures these agents. Matus summarizes this situation so common in social organizations, with the phrase “what is planned is not done, and what is done is not planned.”^2^^,^^3^^,^^4^

The two preceding paragraphs provide empirical and theoretical basis on the problem addressed in this article, which focuses on the State social institutions, encompassed by Bourdieu under the concept of “the left hand of the State.”^5^

The main concepts we work with are: play, practices, and micropolitics. We understand play as a form of freedom “foundational of culture,” based on the idea of *Homo ludens*,^6^^)^ and as a “guiding thread of ontological explanation” in Gadamer terms,^7^ in relation to the concepts of desire and immanence.^8^^,^^9^^,^^10^^,^^11^ From Michel Foucault, we adopt the concept of practices, defined by the regularity and rationality accompanying different modes of doing.^12^ The concept of micropolitics is explored following the developments of Deleuze and Guattari, for whom micropolitics are devoid of conditions, pure becoming on the plane of immanence and relationships, as living work.^10^^,^^11^^,^^13^^,^^14^ These concepts, transformed into stakes, have all the potential of the event.^9^

This essay continues the reflection on the topic of the play developed in previous texts,^14^^,^^15^ and its aim is to analyze play as a central element in the practices that shape the micropolitics in the State social institutions.^14^ The hypothesis is that the size of the organization is inversely proportional to the development of play.

### The play in modernity, another epistemological omission

In both Antiquity and the Middle Ages, plays constituted the social life of people and their processes of socialization.^16^ It is during modernity that play was limited to recreational issues of childhood and outside the idea of work, thus losing its playful dimension, with serious consequences for institutions, their workers, and users.^17^ Why, if there were records from ancient times of the importance of play as a social and socialization phenomenon, was it limited to childhood from the 19th century onwards? The answer is to be found in the Industrial Revolution, which, together with England’s imperial vocation and Victorian morality, constituted a new order, in which economic thought introduced the idea of utility^6^^,^^18^ and piecework.^19^^,^^20^ In this logic, play, being considered unproductive and a waste of time, was annulled, and its place taken by different rationalities that invalidated playful, formulating universal models that, disdaining practices, reduced communication to writing.

*Homo sapiens* came to represent the rational, and its orders were to be fulfilled by *Homo faber*, who executed them on a submissive object, “the thing”. In modernity, the Industrial Revolution replaced artisanal work, the machine became synonymous with progress, and social processes were mechanized, dehumanizing the ontological nature of the social. In all this, play was annulled.

Modern reason was based on the relationship between the thinking (*res cogitans*) and the external thing (*res extensa*), seeking mechanical models as a solution to problems, models that were applied to the social from positivism. The factory^19^ and the machine man ^20^ became dominant ideologies, to which practices, including social ones, had to be subordinated, understood as linear, mechanical, and repetitive. Thus, other epistemologies were annulled, and the social was interpreted from a logic of subject-object relations, omitting the question of being. And in this “forgetting”, language,^21^ play,^6^ and relationality^22^ were annulled, omissions that ended up denying otherness.

For Deleuze and Guattari, “the subject and the object provide a bad approximation of thought. Thinking is not a thread stretched between a subject and an object, nor a revolution around another. Thinking rather occurs in the relationship between territory and land”.^23^ The conceptions of modern reason have dominated and continue to dominate university curricula, which, in general, are replicated by their graduates, regardless of their disciplinary training, age, gender, or political identity. It is this modern reason that highlights the epistemological inadequacies of individuals to play the social play. This becomes visible when moving from “should be” to action, and faces relational processes based on language and play that, at best, they can describe but cannot play. Faced with this reality, they tend to fall back on the instrumental, seeking tools they imagine will solve problems, and repeat the question “how to do it” ignoring that “knowing how” expresses a means and not a culture. Confusing these dimensions leads to the objectification of the other.^24^ The search for tools to provide answers on “how to do?” refers to the idea of an explanatory totality characteristic of modernity, which annuls the subject, thought, action, the multiple, and the uncertainties that constitute the social play. Despite the failures of instrumental reason in solving social problems, the agents do not consider changing the questions and asking themselves, for example, about the whys and wherefores of what they do and what they think.^25^^,^^26^^,^^27^

The logics of “should be” that dominate teaching in universities fail in the presence of the social play, which opens the door to the entry of Sartrean irony: “hell is the other”, which becomes the dominant explanation for failure.^28^ The complexity of the relational, of language, and of conversations, so important for social institutions, finds no support in modern reason, which only aims to describe what it observes and takes that description as “real”, as the only possible truth, based on studies, techniques, and decontextualized methods that draw on positivism, and on unfeasible diagnoses, as they annul otherness, play, and conversations.

General management theory, from its beginnings, between the late 19th and early 20th centuries, did not take long to identify the complexity of subjects and their relationships and, in its evolution, increased investments in technologies via informatics, robotization, and artificial intelligence to replace the complexity represented by groups of workers.

Contrary to this logic, social institutions significantly increased the number of workers, as well as the size of their institutions, in which the same regulations and logics of the industry were applied, since social institutions were considered by the GMT as another factory.^29^

The founding premises of the GMT were to conceive the working person as *Homo faber*, to interpret the organization as a pyramid, to understand the institutional as a combination of the rational and the material, and conceive management and governance places as spaces of high concentration of power and rationality. However, the evolution of the institutions of the social fields presented radical differences with the industrial vision of GMT. These differences are based on the fact that social fields are structured in relational logics, centered on language and play. Nevertheless, social organizations continue to be thought and conceived as if they were dominated by subject/object logics disconnected from the territory. These ambivalences are not gratuitous for workers or users of social services. Dominant ideas cannot accept that organizing necessarily implies reorganizing^30^ and refounding.

## THE RETURN OF PLAY

The annulment of play was challenged, during the 20th century, by different intellectuals who emphasized its relevance, such as Jean Piaget, Melanie Klein, Donald Winnicott, Lev Vygotsky, Jacques Derrida, and Pierre Bourdieu.

Play, in general, is not the object of the study, but we do find it in many texts, in expressions such as “*what is at stake*” or “*getting into the play*”. In this section, we will work on the idea of play as a foundational form of freedom for culture,^6^ as “the guiding thread of ontological explanation”,^7^ and in its relationship with immanence and desire.^10^^,^^11^

### Play as the foundation of culture

In 1938, Johan Huizinga^6^ recovers play as an object of study for the sciences, and in his book “*Homo Ludens*,” he carries out “a genealogy of culture in relation to play,” which he understands as:

“...a free action or occupation, carried out within certain temporal and spatial limits, according to absolutely binding rules, although freely accepted, an action that is its own end and is accompanied by a feeling of tension and joy and the consciousness of ‘being otherwise’ than in ordinary life.”^6^

The idea of play exceeds the human and includes the animal world, producing a narcissistic wound to *Homo sapiens* while simultaneously nullifying any rational foundation of the playful.^6^ For Huizinga, it is through play that one learns,^6^ and it is in playing that we find the origin of all culture. “Why and for what purpose do we play?” Huizinga asks, and answers, that the passion and intensity placed into play has no biological explanation and is incomprehensible to reason.^6^

Play opposes seriousness because seriousness is structured, and every play is, by essence, a free activity that produces the sensation of feeling at ease.^6^ Huizinga finds that in the subject´s devotion to play, it surpasses banter, logic, and biology, and he emphasizes that it cannot be understood as a mental form.^6^ We play because we feel pleasure doing so, as we experience it as an expression of freedom, as something that allows us to escape from routine. Play is not an expression of disinterest, but is culture, which often goes beyond interest and is enclosed in itself with limits of time and space.^6^

For Huizinga, play, while creating order, is order, and requires order to be played:

“...the play oppresses and liberates, the play captivates, electrifies, enchants. It is full of the two noblest qualities that man can find in things and express them: rhythm and harmony. [...] The play tests the player’s faculties: their physical strength, their endurance, their inventiveness, their daring, their endurance, and also their spiritual strength, because, in the midst of their ardor to win the game, they have to stay within the rules, within the limits of what is allowed in it. [...] Each play has its own rules.”^6^

Culture is always played under an unspoken agreement of rules, and as long as the play remains pure, the character of playfulness is maintained. But the more complex the play, the greater the nervousness and uncertainty for the players about what is at stake.^6^ The development of a culture itself means that the play does not remain unchanged and produces an almost imperceptible transition from the play to the sphere of the sacred, marking the continuity in a triple union that occurs between play, celebration, and sacred action.^6^

The power of playfulness erodes the bases of reason and tradition. In Huizinga’s terms, just as “almost everything abstract can be denied, for example: law, beauty, truth, goodness, spirit, God, and seriousness. Everything can be denied, but play cannot.”^6^

### Play as a thread of ontological explanation

The title of this section corresponds to one of the chapters of “Truth and Method” by Hans-Georg Gadamer.^7^ Play occupies a significant place in his work, and he considers it a natural process, not exclusive to humans, as animals and nature also play.^7^ For Gadamer, play is never a mere object, but exists for those who participate in it.^7^

The German word “*spiel*” translates into Spanish as “juego,” but in its translation, it loses a series of semantic associations that it has in German, as pointed out by the translators of Gadamer’s book. The original meaning of the word “*spiel*” in German is dance, which is preserved in some compound terms as “*spielmann*” (minstrel). It is also associated with the world of theater: a theatrical piece is a “*spiel*” (play), the actors are “*spieler*” (players), and the play is not performed, but played (“*wirdgespielt*”).^7^ Gadamer points out that play goes beyond the consciousness of the player and, although it refers to the subject’s experience, it is distinguished from the subjective behavior of the player.^7^

For *Homo sapiens*, play is not considered something serious; it is seen as a distraction, and it is believed that play is just that, a play. However, a player who does not take the game seriously and undervalues it is considered a spoilsport.^7^ When is play truly play? “When the player completely surrenders to the game,” answers Gadamer. He believes that the definition of what play is should not be sought in the reflection of the player, nor as an expression of subjectivity, but in “the mode of being of the game itself,” and to do this, one must eliminate the idea that play exists within the rationalities imposed on institutions in the social field.^7^

For Gadamer, the question should be directed towards “the mode of being of the play itself,” which leads him to recognize that “the subject of the play is not the players,” but rather the play, which manifests itself through the players in “linguistic processes that constitute linguistic plays”.^7^ The fascination of the play lies precisely in stepping out of consciousness to enter a context of movements that develop their own dynamics. The play refers to a movement without objectives, to a continuous present, which is recreated in constant repetition, regardless of who performs it, to the extent of dispensing the person when expressing “something is at stake” in a dialogue. This centrality of the play allows the player to surrender to it and to detach from the initiative that “constitutes the true effort of existence,” which makes understandable the impulse to repetition in the player and the fear of changing the play.^7^

The player only requires another who plays and responds with their counter-initiatives.^7^ That other may not necessarily be a real player; a current example of this is the technological gamification of games. According to Gadamer:

“All playing is being played. The attraction of the game, the fascination it exerts, lies precisely in the fact that the game takes possession of the players. Even in games where one must fulfill tasks that one has set for oneself, what constitutes the attraction of the game is the risk of whether ‘it will be possible,’ ´whether it will come out’ or ‘it will come out again.’ The one who tempts in this way is actually tempted. Precisely those experiences in which there is only one player makes it clear to what extent the true subject of the game is not the player but the game itself.”^7^

The world of games remains closed to the world of objects. Playing is always representing; the player experiences the game as a reality that surpasses it. In the game, there are no references to spectators and, if the game becomes a competition, it threatens its playful character.^7^

Play is transformed into the action of playing and, therefore, what arises as unforeseen and improvised can become repeatable, and can even become permanent. Hence the constructive character of play, which can become a work. These ideas conflict with the dominant ideas in the sciences, which demand understanding the game and therefore require the subject to stay outside the game, a process by which the playful is replaced by the rational, creating experts, commentators, and critics who share in not playing the game. But the game needs players and, in this contradiction, the university and its members often become trapped.

### Play, immanence, and desire

Nietzsche hierarchizes play, as an activity prior to the implementation of forms, thereby breaking with the traditional scheme of knowledge, as there is no *a priori*, given that play creates and anticipates forms.^16^ For Nietzsche, play is a theoretical object, an interpretive scheme and an unconditioned project, which refers to the concept of immanence, as a judgement of the traditions of thought, customs, authorities, and reasoning, which he considers as acquired. In all of this, Nietzsche does nothing more than confront the concept of transcendence as a superior reality dominant in Kantian thought.^8^

This notion of Nietzschean immanence is also present in Eastern culture as a psychic phenomenon that does not transcend it, since it inherently goes together in an inseparably way with its essence, although rationally it can be distinguished from it. The plane of immanence does not engage with the “must be” of rationality formulated from the plane of transcendence. The plane of immanence comes into play in singular bets or agreements to share horizons from which micropolitics emerge, often expressed in playful forms.^7^^,^^9^

Play, as part of work, constructs the present on the plane of immanence, which is considered by Deleuze, following Spinoza and Nietzsche, as a reality superior to the Platonic world of ideas. Deleuze places play on the plane of events, singularities, and intensities.^10^

### All plays, the play

The situations experienced daily in their institutions by workers in social fields are often very uncomfortable and disillusioning, and over time, disillusionment is compounded by anger. This leads them to disengage, to disaffiliate from the institution, as they believe that what they experience is a problem specific to the institution where they work, and that it cannot be fixed. Their own rationality prevents them from understanding the nature of the play in which they are immersed; they cannot consider that play is the ontological quality of work in these institutions and, on the contrary, they demand the rationality of a factory, a reality very away from social institutions.

Those who work in social institutions think and imagine institutional life from a rational perspective, not from play. Gadamer considers that, in the relationship between the player and the play, the player does not know that he knows it.^7^ The question that arises is: where did he learn the play? The answer is straightforward: in the practices.

The lack of awareness of the importance of play can be transformed into knowledge if the teams are encouraged to converse and reflect on what they do every day, but allowing their feet to speak, and avoiding the intervention of their heads with their knowledge, or common sense pointing out the “must be” and annulling the rich and unique experiences of doing, which we associate with the expression “let the feet speak”. This expression, which we have been using for many years, we found in Jacques Lacan, who, in a lecture given at the Massachusetts Institute of Technology on December 2, 1975, in response to Noam Chomsky’s question about his opinion on thought, answered following the existing polemic between them:

“We think we think with our brains, but personally, I think with my feet. That is the only way I can contact something solid. Occasionally, I think with my head when I bump into something. But I have seen enough encephalograms to know that there are no indications of thinking in the brain.”^31^

In workshops we conduct with workers from different social areas, we use a dynamic that consists of each person describing their daily activities in detail, describing what they do, how they do it, and with whom they agree to do it. And as people start to narrate, they realize that their stories have nothing to do with the knowledge in their heads, nor with the organization’s norms, but are spoken by their feet, from their practices. Their narratives surprise them and provoke laughter; they are amazed to discover the contradictions between the ideas in their heads and the movements of their feet that transgress regulations and organizational charts but make the impossible possible.

The above narrative will not be very different from what we can find in people in charge of a management position or the administration of a social institution, if we ask them to relate their day-to-day work, from what they do (their feet, the practices), and not from what they think they should do (their head, the plans). It would be very strange if they express that they rely on planned, programmed, evaluated, and monitored processes, which accompany the fulfillment of orders with accountability of results to their superiors and to the people under their charge. On the contrary, it is very likely that their narratives will refer us to play, even if they do not mention that word, to uncertain results, and it will surely be complex to understand which is the dominant social play. It is also very likely that their stories will go through different examples that allow us to associate them with a dance; to an amusement park with bumper cars, ghost trains, or roller coasters; to an Olympic Games with 4x100 meter relay races, where the baton is rarely passed successfully; to swimming competitions, but unsynchronized; to wrestling or target shooting scenarios; to performing arts with tragedies, comedies, tragicomedies, dramas, or monologues; or, simplifying, they end up associating it with the “Antón Pirulero” where “each one attends to its own play...”.

What those who narrate their day-to-day experiences will surely recognize is that the scenarios in which they developed their play are not part of their university education. They will also recognize that when they arrived at the position, they did so full of plans, goals, and objectives, all of which had in common a disregard for the social play. And over time, they discovered and understood that everyone plays in a continuous and uncertain process of great learning, not without frustrations, but also not without joys. Nor did it assume the complexity of the problems they had to face and the chaotic sequences that arose in their agenda, dismantling what was planned and generating urgencies, which were not always such. They also discovered the complexity of communications and that the Tower of Babel is not just a biblical story. They learned that miracles exist and, without knowing why or how, sometimes problems were solved without their intervention.^32^

Administrative rationality tries to impose on social organizations ways of doing things following rules, which nullify conversations and play, ignoring the fact that these teams, when they achieve the sense and meaning of their duties, transform the institution into a playroom. The narratives of people who work in, lead, or manage social institutions are very different from those who do so in industrial institutions, where rationality tends to dominate. This does not mean idealizing them and thinking of them as free of conflict, but rather recognizing them as institutions that are more predictable than social institutions.

## GOODBYE TO THE PYRAMID

The ontological character of the work process in social institutions is artisanal, since the coordination mechanism is achieved through “mutual adjustment,” which triggers a domino effect on the logic that structures the GMT.^13^^,^^14^ This has an impact on the organizational form, which becomes to resemble a professional bureaucracy,^33^ given the high power that exists at the base of the organization - operational cores - where the artisanal work of professionals resides.^33^ The most evident examples of professional bureaucracies, according to Henry Mintzberg, are universities, hospitals, schools, artisanal production companies, research-oriented institutions, and architecture schools, among others.^33^ Professional bureaucracy affects institutional forms, given the centrality and relevance of language and conversations in the work, transforming organigrams and missions formulated from functionalism into museum pieces. Management and governance also change, as the aforementioned transformations imply a singularity and complexity unknown to GMT for those managerial positions.^2^^,^^29^^,^^32^

These institutional dynamics that occur outside of rationality are played daily, leading social institutions to fragment like archipelagos, which conceal themselves with denominations that evoke the idea of a unique, strong, and consolidated institution under the name of school, college, university, or hospital. These names represent nothing but a fragile scenography, revealed upon entering the institution and recognizing the different plays, players, conflicts, and alliances in unpredictable dynamics, leading these archipelagos to multiply and reconfigure in unforeseeable ways. This indicates that, in social institutions, the pyramid is in the players´ head and not in their plays.

### The factory imprint on the materiality of the social institution

Social institutions uncritically adopted the materiality of the factory institution in different social fields, leading to the creation of large institutions. The question we ask is: What is the advantage of having large institutions in social areas? The hypothesis we uphold is that the size of the organization is inversely proportional to the development of play in the practices. This should not be simplified by assuming that reducing the size of organizations solves all their problems, as they are hypercomplex institutions,^34^^,^^35^ where the size of the organization is a central element of this complexity, but not the only one.

The ambition of the “big factory” is found in different social fields and continues to seduce political actors who are captured by pharaonic projects and ostentatious inaugurations, but whose social effectiveness is highly questionable.

We ask ourselves: Is it the inexorable destiny of social institutions to become total institutions? Will we dare to think and implement other institutionalities? Will we accept that large institutions nullify the play? What is the problem of having small, human-scale institutions that understand the differences and singularities of their populations instead of trying to homogenize them?

Melossi and Pavarini,^36^ in their book *Prison and Factory*, analyze how the prison adopted an institutional model influenced by the factory, allowing it to operate as an instrument of power and social control, perpetuating discipline and its coercive power,^37^ consolidating the prison system as a place of intersection between poverty and race.^38^

The model of the large social institution is also found in the sadly famous institutes for minors, created in Argentina in 1931, under the jurisdiction of the National Children’s Board. In 1972, Enrique Medina described these institutes in his novel *Las tumbas* (The Tombs), a name given by the interns themselves for the institutes for minors. In this novel, the author narrates autobiographically his admission to the juvenile institution when he was in second grade of primary education, and his experiences in various juvenile institutions over ten years. Rodolfo Walsh, in the presentation of the book, said:

In this world, there is almost no other way out other than the transition from victim to victimizer through a long chain of simulation and submission [...] a rotten society that confines children in concentration camps [...] a vigorous and surprising testimony about a category of social prisoners.^39^

Goffman, in his work *Asylums: Essays on the Social Situation of Mental Patients and Other Inmates*, highlights how psychiatric institutions affect the identity and social interaction of individuals, becoming total institutions characterized by blurring the boundaries between the different roles within the institution. Basaglia describes psychiatric hospitals as a “denied institution,” considering them oppressive and dehumanized institutions, despite the existence of physical and organizational structures that claim to be places of treatment and care when they actually deny the needs and fundamental rights of people with mental illnesses. In his view, these institutions perpetuate segregation, stigmatization, and mistreatment towards patients.^40^

Basaglia was convinced that mental hospitals were institutions that could not be reformed and that it was necessary to abolish them to restore freedom to the patients and transform them into support centers. Until then, they had been places of confinement, inhumane treatment, and forced medication. He proposed the self-determination of the institutionalized persons to reintegrate them into a dignified life based on his main motto: “Freedom is what heals.”^41^

## THE GREATNESS OF SMALL INSTITUTIONS

Faced with a social reality that has become increasingly complex, we advocate for small institutions in terms of size, with a rhizomatic configuration, in order to safeguard the micropolitics that humanize institutional life.^10^ We understand the social institutions of the State as a social network, not technological but human, centered on conversations grounded on the commitment between what is said and what is done,^42^ with the construction of agreements to share horizons^7^, with a strong imprint of the symbolic, and with objective evidence of the impact of actions in the territory.

It is necessary to build social institutions of the State on a human scale, away from any vestige of industry, and conceived from relational logics, with the importance and complexity of the relational. Among the qualities that we consider necessary to configure another institutional framework, we highlight the need to institutionally integrate social policies in the territory; prioritize social and cultural ties; humanize care; recognize the diversity of identities, ethnicities, and cultures; and promote collegial management with the inclusion of territorial actors.^43^^,^^44^^,^^45^^,^^46^^,^^47^

We advocate for other institutionalities, not because it is easy to achieve consensus on this proposal, but because we consider it is very necessary to do so, which is not synonymous with easy. Human-scale institutions favor plays, practices, and micropolitics. But if institutions grow, inevitably, they begin to be invaded by bureaucratic procedures, with the consequent depersonalization of processes that end up nullifying plays. It is then that the play becomes serious, and with the disappearance of the playful, arboreal dynamics with Kafkaesque elements are strengthened, leading to disaffiliation of the public, not only in workers but also in the collectives of the territory that stop feeling those State institutions as their own and begin to hear siren songs promising them what they will not give them, but that public institutions also do not provide. With the withdrawal of the plays, the hierarchy of the relational is devalued, norms appear, and communicative action becomes technified.^48^

We are talking about other social institutionalisms, not new, since what we are discussing revisits experiences that were ignored or displaced by the advance of industrial and technological models that dominated and still dominate the thinking about institutions and their organizational forms. This whole process will necessarily involve the division of many existing institutions into smaller ones and the elimination of certain institutional forms such as asylums and nursing homes, without any worker losing their work. The firm purpose remains to humanize relationships and create senses of belonging between territories and institutions.

And what about central levels? They should undergo a strong reconfiguration that leads them to abandon their *habitus* as planners and prescribers of “should be”, to become smaller but smarter and more agile institutions, focused on monitoring territorial dynamics and actions. They will be supported by robust and dynamic information systems that, through integrated analysis, produce information about actions, processes, and results, which should be discussed in the territory with the populations, from perspectives that integrate the social aspect and go beyond disciplinary knowledge with actions that seek effectiveness through the comparison of disaggregated results at different local and regional levels, always respecting territorial singularities.^14^^,^^49^^)^ The aims is to unleash processes that allow for analysis, projections, and interventions on territorial issues and problems, undertaking compensatory actions and respecting idiosyncrasies in processes of knowledge translation.^50^ All of the above should be part of transparent and freely accessible systems of request and accountability .^2^

The proposed changes will be resisted, since they will affect political, economic, labor union, and institutional cultural interests. These transformations are easy to write on paper, but very complex to implement, because they cannot be solved with decrees or laws. Therefore, we argue that this entire process must be accompanied by a strong publicity to transform the model of the macro-social institution into a public issue that is socially debated,^51^^,^^52^ with the purpose of gradually building consensus on alternative institutions. Nevertheless, the journey will be very arduous, as it is not a scientific discussion but basically a political discussion in which strong interests are at stake.

### From economic scales to human scales

In the industrial world, one way to classify institutions is based on size (large, medium-sized, and small),^29^ by the volume of their assets, or by the number of employees. These classifications follow economic scales that should not prevail in social institutions since, as the popular saying goes, “you get what you pay for.”

There is a rich discussion about the size of institutions. From the critical theory of the Frankfurt School, different authors have pointed out that the size of institutions can shape human experience and society. Mega-institutions not only affect the effectiveness and internal structure of those institutions but also impact freedom, individual identity, democratic participation, and social dynamics. The main contributors of this school analyzed the relationship between rationality, domination, and social organization, finding that modern rationalization and bureaucratic organization lead to forms of domination and alienation.

Adorno and Horkheimer argued that massive organizational structures generate a system of control that inhibits freedom and human authenticity, turning individuals into mere gears in the social machinery.^53^ Herbert Marcuse pointed out the alienating consequences of industrial society through modern, increasingly large and centralized organizational forms, which limit individual freedom and tend to promote a conformist and alienating mentality, where individuals adapt to predefined roles and lose their capacity for critical thinking.^54^ According to Habermas, the size of an institution can influence the public sphere and decision-making, arguing that massive organizational structures can distort communication and hinder democratic participation, affecting the formation of opinions and public discussion.^48^

Ernst Schumacher, a German intellectual and economist, who served for two decades as Chief Economic Advisor to the National Coal Board of Great Britain, in his book *Small is Beautiful: Economics as if People Mattered*,^24^ published in 1973, criticizes the orientation of societies focused on economic logics, a view also followed by science and technology, to the detriment of people. One of the chapters is titled “A Problem of Size,” in which he analyzes the growth of large companies and how some of them have created the small within the large, trying to achieve a balance between freedom and order, where freedom is expressed in small units and order in a global organizational unit. Schumacher asks:

What scale is appropriate? It depends on what we are trying to accomplish; the problem of scale is extremely crucial today politically, socially and economically. The idolatry of gigantism, which I have already spoken, is possibly one of the causes and certainly one of the effects of modern technology. What is the meaning of democracy, freedom, human dignity, standard of living, self-realization, and complete satisfaction? Is this a matter of commodities or of people? Of course, it is a matter of people. But people can only be people in sufficiently small groups. Therefore, we must learn to think in terms of an articulated structure that can accommodate a varied multiplicity of small-scale units. If economic thought cannot grasp this, it is entirely useless. If it cannot rise above its broad abstractions, such as national income, growth rate, capital/product ratio, input-output analysis, labor mobility, and capital accumulation; if it cannot rise above all of this and make contact with a human reality of poverty, frustration, alienation, despair, demoralization, crime, escapism, tension, congestion, deformity, and spiritual death, let us set aside economics and start again. Do we not already have enough “signs of the times” indicating that we need to start over? ^24^

The ideas contained in the preceding paragraph help us argue that social institutions should be small, with a logic of reproduction characterized by rhizomatic nature,^10^ thus challenging the dominant ideas of factory models erroneously but not innocently adopted by social areas. These models raised to the creation of large hospitals, schools, universities, courts, and prisons, among the most well-known examples, which have dominated the institutionalism of the social sphere. Which in their hegemony, disdained the small institutions as an expression of low professional and scientific quality.

Norbert Elias poses the question: when is there more conflict in an organization? And he arrives at the following conclusions: when there are many workers, when they do not know each other, and when they do not know what others are doing.^55^ thus demonstrating the obvious, recognizing that the social is essentially relational.

We aspire to small institutions with a limited number of workers, more humanized than technified, and focused on caring for others. Institutions concerned with incorporating “players” rather than spectators, narrators, or commentators, where the playful dimension of working while playing prevails. Building plays without winners or losers, where pleasure arises from playing, where humanization prevails over objectification, social rights over the market, caring over healing, and learning over teaching.

We take the expression “*Quillahue*” from the *Mapudungun* language - which means a place that helps - to ask ourselves whether these other social institutionalities can become places that help.

### Institutions in the field of education

Teaching and learning are central concepts in education, but they imply very different conceptions. Teaching assumes that there is someone who knows and someone who does not and, therefore, the one who does not know must be taught. This conception of education is considered by Paulo Freire as banking education, since it conceives learning from the passivity of being in the classroom, sitting on the bench. A very different situation from the idea of learning, which is based on the premise that everyone knows something, and that each person will find the time and the best way to learn, in a process mediated by action, which does not disdain play. Thus, while teaching assigns to the teacher the central role, in learning, the teacher accompanies the student’s initiative, who in their search learns from the question, while working and playing.^56^^,^^57^

John Dewey, in the United States, between the late nineteenth and early twentieth centuries, emphasized learning based on the experience and student participation in small environments, where interaction between students and teachers was as close and personalized as possible, highlighting the importance of practical experience and social interaction.^43^ Abraham Flexner developed successful experiences, also in the United States, following the postulates proposed by Dewey at the college,^44^ and argued that the quality of the university was measured neither by size, nor the number of programs, nor the number of students.^58^ Similar experiences occurred in the early twentieth century from anarchism, led by Francisco Ferrer Guardia from Barcelona.^59^^,^^60^

Different educational theorists highlight the importance of personalized interaction, collaboration, and active learning, which are easier to achieve in small, student-centered educational environments.^57^^,^^61^^,^^62^^,^^63^^,^^64^^,^^65^ Francesco Tonucci, an Italian pedagogue, argues:

Paradoxically, we could affirm that those who do not need it succeed in school. The school, which should contribute to introducing equality among citizens, on the contrary, contributes to nurture differences [...] The teacher is not the knowledge but the mediator of knowledge [...] The transmissive school assumes that the child does not know and goes to school to learn, while the teacher teaches those who do not know. This is a childish idea, which thinks about the child as an empty vessel, while the teacher pours knowledge that gradually fills the child [...] The child knows and is competent and goes to school to develop its knowledge.^65^

Small educational institutions can facilitate more personalized attention and greater possibilities for interaction with teachers, creating a stronger, more collaborative senses of belonging and facilitating more practical and personalized learning. Different current publications point out the benefits of choosing for small schools.^66^^,^^67^^,^^68^^,^^69^

### Another institutional hegemony in the field of health

Schumacher proposes a series of principles applicable to organizations:

The first principle is called The Principle of Subsidiarity or The Principle of Subsidiary Function. A famous formulation of this principle reads as follows: “It is an injustice and at the same time a grave evil and an outrage against the order to assign to a larger and higher association what smaller and subordinate organizations can do.”^24^

We will rely on this first principle to argue that self-care,^70^^,^^71^ other non-medical rationalities,^72^^,^^73^ and primary health care centers are capable of resolving at least 90% of people’s illnesses. This scientific evidence was generated by Kerr White in 1961 in his work on the ecology of medical care,^74^ with data from the white population of the United States from the period 1928-1931 and population data from England and Wales from the period 1946-1950, which has been replicated in numerous publications since then. In an analysis of studies on the ecology of medical care that we recently published, based on nine research studies conducted in the United States, Japan, Canada, Austria, South Korea, Israel, and Austria between 1996 and 2018 at the country level, we found very similar results to Kerr White’s work,^75^ indicating a regularity over a period of 90 years that must be analyzed and discussed, as it does not correspond to the medicalized ideology of modern societies.

Primary health care centers have been denied an institutional character that would give them a hierarchical status, using different expressions which combine childish phrases with diminutive expressions, or erratic denominations such as: “the little health center,” “the health post,” “the first aid room,” “the peripheral center,” or “the dispensary.” All these expressions imply institutional undervaluation and, indirectly, an overvaluation of the figure of the hospital, which has a univocal and indisputable nomination. Another way to delegitimize primary health care centers was to force general practitioners or family doctors to only work on emergencies in hospitals when, due to their specialization, they should work on emergencies at primary health care centers. All these symbolic and semiotic aspects should not be overlooked in the debate over the prioritization and legitimacy of primary health care centers.

During the 20th century, there were numerous successful experiences of primary health care centers, but few of them lasted over time, unlike hospitals which, wherever they were created, not only remained but also grew at levels that were -most of the time- unjustified from the perspective of epidemiology of health services and systems.^76^

Evidence of the importance and usefulness of primary health care centers close to population can be found in the United States from the late 19th to the early 20th century.^77^ Winslow, in 1919, stated that primary health care centers were the most notable event in the evolution of public health in the United States.^77^ Michael Davis, in 1927, made similar statements in the same vein.^14^^,^^78^

The *Dawson Report*,^79^ published in England in 1920, is considered a central document for what would later become the National Health Service (NHS). It refers to three institutional levels: primary health care centers, secondary health care centers (hospitals), and teaching hospitals. More than 100 years ago, the Dawson report established what should compose a primary health care center; its reading will not fail to surprise the reader:

In the primary health centers, would be gathered together the health services and activities of the districts [...] There would be wards of various sizes and for different purposes, including obstetrics. The increasing employment of open-air treatment of illnesses would be provide for. [...] Further accommodation might include the following: Operating room, with the necessary equipment. Radiology rooms. Laboratory for simple investigations. Dispensary. Baths, including simple hydrotherapy. Equipment needed for massages, electricity, physical culture [...] Communal services [...] There would be accommodation for communal services such as those of prenatal care, child welfare, medical inspection and treatment of school children, physical culture, examination of suspected cases of tuberculosis and occupational diseases, etc. [...] So far as midwifery services were not available in particular districts under other arrangements , their services could be provided from the Center and the Center residential accommodation could be found , not only for nurses and midwives working there, but also for those engaged in rending similar services in the neighborhood. [...] An important aspect of the center would be a dental clinic, of visiting dental surgeons, employed either part-time or, where necessary on a whole-time, and nurses attached to the service [...] The primary center would be the home of the health organization and intellectual life of the doctors of that unit. Those doctors, instead of being isolated, as now from each other, would be brought together and in contact with consultants and specialists; there would develop an intellectual traffic and a camaraderie of the great advantage to the service. No doubt discussions and postgraduate instruction would in time be organized, and Study leave to teaching hospitals could easily and advantageously be arranged. It would provide all ordinary forms of treatment to patients of all ages [...] the work of the general practitioner would be mainly domiciliary but partly institutional, mainly individual, but partly communal.^79^

In 1946, John Grant, an official of the Rockefeller Foundation, suggested that the health center of the future was about to be created, referring to primary health care centers.^14^^,^^77^ In 1952, Henry Sigerist, in a lecture at the London School of Hygiene and Tropical Medicine of the University of London, emphasized the importance and necessity of primary health care centers.^80^^,^^81^

In Latin America, there is a long list of experiences of different scales and at different times and countries, but generally, they are short-lived. These experiences are the result from reflections of individuals whether from professional or political areas, with humanistic values, or from political situations in revolutionary processes.

In recent decades, especially in Scandinavian countries, progress has been made in transitioning from hospital-centered care to primary health care.^82^^,^^83^

The *Dawson Report*, 104 years after its publication, is clear evidence of the serious institutional error that was made by prioritizing secondary health care centers (hospitals) over primary health care centers.

In 2017, Henry Mintzberg, in his book on myths in healthcare management, points out that:

In healthcare institutions, especially hospitals, scale receives considerable attention from administrative engineers, who deal with problems by trying to make them bigger […] As institutions grow, they overlay one level of administration over another in their formal hierarchies. We have already discussed what can be called the politics of scale: the inclination to merge smaller hospitals into larger ones by the power of administrations. This can also be equivalent to convenience of scale: central administrations take time and effort to deal with numerous small institutions-hours upon hours of their time. How much more convenient it is to group them all and deal with one, even if that may result in many service providers being miserable for years. Each of them, being increasingly removed from operations, relies more and more to reorganization, reengineering, measurement, and other simple solutions […] Additionally, large scale can impose a certain conformity on activities. This can make them easier to handle, but it can also suppress needs for local adaptation […] Small institutions often can address certain problems in less formal and more effective ways. For nearly everything about healthcare (and beyond), the imperative that scale, measurement, leadership, etc., to be some sort of “one best way” is simplistic and dysfunctional. Traveling down the medical production line is not a chassis or an engine; it is ourselves. […] Especially when we are sick, but even when we are well, we often find large institutions to be impersonal and alienating. That can influence the effectiveness of the treatments we receive. It is no surprising that there has been a shift away from large hospitals, much like from large schools. To achieve real quality in healthcare, we need personalized services on a human scale, not impersonal interventions on an economic scale. I am not saying here that small is always beautiful; I am only asserting that bigger is not necessarily better […] The scale problem seems to apply to pharmaceutical research as well. Some of the largest companies have been facing difficulties in their research for years. An executive at Merck, the company most recognized for its research, commented that “scale has not been an indicator of the ability to discover innovative drugs. In fact, it has been the opposite: one gets stuck.” […] Much of the interesting pharmaceutical research now comes from smaller companies.^84^

### The prison institution

The prison, in its classic institutional form, has low chances of producing social reintegration.^85^ The discussion on the size of prisons has been the subject of attention by academics, activists, and professionals in the judicial system, who have questioned whether smaller prisons can be more effective for rehabilitation by theoretically allowing greater individualized attention, more adapted reeducation programs, and closer relationships between staff and inmates. Smaller prisons might be more expensive from an economy of scale logic; however, the additional costs may be worthwhile if they lead to better rehabilitation results and lower recidivism rates. Multiple voices^86^^,^^87^^,^^88^^,^^89^ argue for a more individual-centered approach and less dependent on large facilities, emphasizing the quality of rehabilitation over the quantity of inmates.

## END OF THE PLAY OR OTHER INSTITUTIONALITIES?

For decades, we have witnessed historical moments dominated by a primacy of the individual and the private, to the detriment of the collective and the public. This situation, pointed out since the second half of the 20th century by different authors,^90^^,^^91^^,^^92^^,^^93^^,^^94^^,^^95^ was a task deepened by neoliberalism worldwide in recent decades and has been expressed in governments, in different countries, with antidemocratic characteristics, aiming to dismantle social rights and public institutions in the name of market freedom.

In recent decades, new subjectivities have emerged that reject the social institutions of the State and the idea of the Nation. These new subjectivities have also been reproduced in social sectors historically abandoned by State policies and suffering situations of social exclusion or extreme poverty.^96^

The resignation or withdrawal of the State, the reification of individuals, the construction of self-centered subjectivities, social exclusion, and the crisis of legitimacy of the public sector constitute a very complex reality, which is exacerbated by the progressive growth of social debt.

The neoliberal horizons in subjectivities foreseen by Jorge Alemán, in less than ten years have become very serious political realities in Argentina. The debates on democracy, the State, and citizenship which took place in Latin America from the 1980s onwards, seem very distant.^97^^,^^98^^,^^99^^,^^100^ The current times indicate that these topics still constitute significant debts in Latin American societies, debts that could not or would not be settled.

The rupture of social relations, the dominance of aporophobia, and the abandonment of any project to reduce inequalities challenge the social institutions of the State, its policies, and actions in the territories. Bourdieu, in the 1990s, in France, left testimony about the resignation of the State,^96^ expressed in the absence of the right to the State and citizenship for the majority of the population, and proposed the concept of “the left hand of the State” to indicate how the impoverishment of large social sectors in France complicated the work of the State’s social institutions and the impact of these new realities on subjectivities.

Faced with this reality, it is imperative to put on the agenda the need to produce cultural and structural changes in the social institutions of the State, in order to recover their social function and provide quality public services, from the territories. It is there, in the territories, that these institutions become one of the “faces” of the government, that is, they become important intermediaries between governments and the population. Reversing these characterizations partly exceeds the possibilities of those who lead a national, provincial, or municipal government, since social areas, due to their characteristics of professional bureaucracies,^33^ can either work miracles or become self-centered, disaffiliating from the public and sabotaging policies originating at central levels.

The challenge facing those who govern, those who work in public institutions, and the numerous social groups in the territories is to care for that institutionality, which must prioritize rights, especially for those who need them most, and to provide the best possible service. Both civil servants and workers should recover with pride the concept of public servants, to generate a work culture that accounts for the otherness of the users. It is useless to feel committed to social causes if practices focused on “what should be” are maintained, denying playfulness and otherness.

We must not accept the end of the play; we must inhabit small institutions where play builds micropolitics in practices that support and care for those who need it the most.

Let’s play!
